# Implementation of a health education programm for teenagers affected by systemic lupus erythematosus

**DOI:** 10.1186/1546-0096-12-S1-P133

**Published:** 2014-09-17

**Authors:** Marie Bucchia, Emma Allain Launay

**Affiliations:** 1 Pédiatrie CHU nantes, Nantes, France

## Introduction

Lupus is a chronic disease for which health education is an integral part of the treatment. Health education consists in teaching the patients how to manage everyday life with their illness.

## Objectives

This study aimed to evaluate the benefits of health education programs among patients with lupus.

## Methods

This experimental study was conducted by two doctors experienced in health education programs, one intern in paediatrics, and two trainee nurses. The health education program was offered to ten young women suffering from systemic lupus erythematosus, aged between 13 and 17 years old. It did not receive any financial support or benefit from additional human resources, and relied exclusively on the internal organization within the board and among the research team members.

## Results

Seven of the ten women involved accepted to be part of the program. Every patient was first administered an educational assessment test, that was meant to evaluate the patients' already acquired skills as well as their expectations from the teaching. The program was designed to develop such skills as a better knowledge of the disease, adequate self-care and other psychosocial skills (adaptability, capacity to speak about the disease, etc.)

To this day, two group sessions have already taken place and it has been our main concern to build them along with the patients. These sessions, of a duration of 2 hours and 45 minutes each, were scheduled some time away from the assessment session. Some educational tools were created during the sessions (card games, board games like "Time's up", "Photolanguage", a game specifically designed to help expression of feelings around the disease, other educational games and activities). Additional tools are in the process of being built. They include a tryptic about the disease (its physiopathology, its consequences, the future with lupus), which is intended to the use of other young patients.

The patients' satisfaction with the program was assessed individually and collectively.

This experimental program will be running until the end of 2014. It has been submitted to the Regional Health Agency for accreditation.

## Conclusion

By revealing the patients' demand for and satisfaction with the proposed health education sessions, this study has greatly encouraged us to continue this experimental health education program, which will soon be extended

## Disclosure of interest

None declared.

